# Analysis of recent shared ancestry in a familial cohort identifies coding and noncoding autism spectrum disorder variants

**DOI:** 10.1038/s41525-022-00284-2

**Published:** 2022-02-21

**Authors:** Islam Oguz Tuncay, Nancy L. Parmalee, Raida Khalil, Kiran Kaur, Ashwani Kumar, Mohamed Jimale, Jennifer L. Howe, Kimberly Goodspeed, Patricia Evans, Loai Alzghoul, Chao Xing, Stephen W. Scherer, Maria H. Chahrour

**Affiliations:** 1 Department of Neuroscience, University of Texas Southwestern Medical Center, Dallas, TX 75390 USA; 2 Eugene McDermott Center for Human Growth and Development, University of Texas Southwestern Medical Center, Dallas, TX 75390 USA; 3 Department of Biotechnology and Genetic Engineering, Faculty of Science, University of Philadelphia, Amman, Jordan; 4 The Centre for Applied Genomics and Program in Genetics and Genomic Biology, Hospital for Sick Children, Toronto, ON Canada; 5 Department of Pediatrics, University of Texas Southwestern Medical Center, Dallas, TX 75390 USA; 6 Department of Neurology, University of Texas Southwestern Medical Center, Dallas, TX 75390 USA; 7 Department of Psychiatry, University of Texas Southwestern Medical Center, Dallas, TX 75390 USA; 8 Department of Physiology and Biochemistry, School of Medicine, The University of Jordan, Amman, Jordan; 9 Department of Population and Data Sciences, University of Texas Southwestern Medical Center, Dallas, TX 75390 USA; 10 Lyda Hill Department of Bioinformatics, University of Texas Southwestern Medical Center, Dallas, TX 75390 USA; 11 McLaughlin Centre and Department of Molecular Genetics, University of Toronto, Toronto, ON Canada; 12 Center for the Genetics of Host Defense, University of Texas Southwestern Medical Center, Dallas, TX 75390 USA; 13 Peter O’Donnell Jr. Brain Institute, University of Texas Southwestern Medical Center, Dallas, TX 75390 USA

**Keywords:** Medical genomics, Autism spectrum disorders

## Abstract

Autism spectrum disorder (ASD) is a collection of neurodevelopmental disorders characterized by deficits in social communication and restricted, repetitive patterns of behavior or interests. ASD is highly heritable, but genetically and phenotypically heterogeneous, reducing the power to identify causative genes. We performed whole genome sequencing (WGS) in an ASD cohort of 68 individuals from 22 families enriched for recent shared ancestry. We identified an average of 3.07 million variants per genome, of which an average of 112,512 were rare. We mapped runs of homozygosity (ROHs) in affected individuals and found an average genomic homozygosity of 9.65%, consistent with expectations for multiple generations of consanguineous unions. We identified potentially pathogenic rare exonic or splice site variants in 12 known (including *KMT2C*, *SCN1A*, *SPTBN1*, *SYNE1*, *ZNF292*) and 12 candidate (including *CHD5*, *GRB10*, *PPP1R13B*) ASD genes. Furthermore, we annotated noncoding variants in ROHs with brain-specific regulatory elements and identified putative disease-causing variants within brain-specific promoters and enhancers for 5 known ASD and neurodevelopmental disease genes (*ACTG1*, *AUTS2*, *CTNND2*, *CNTNAP4*, *SPTBN4*). We also identified copy number variants in two known ASD and neurodevelopmental disease loci in two affected individuals. In total we identified potentially etiological variants in known ASD or neurodevelopmental disease genes for ~61% (14/23) of affected individuals. We combined WGS with homozygosity mapping and regulatory element annotations to identify candidate ASD variants. Our analyses add to the growing number of ASD genes and variants and emphasize the importance of leveraging recent shared ancestry to map disease variants in complex neurodevelopmental disorders.

## Introduction

Autism spectrum disorder (ASD) is a neurodevelopmental condition with a current population prevalence estimated at 1 in 44 individuals in the USA^1^. Growing evidence suggests that ASD encompasses a collection of individually rare disorders that share the core features of difficulty with social and communication skills and stereotyped or repetitive behaviors and interests^2^. ASD varies in the severity and presentation of the core symptoms as well as the associated comorbidities, which include intellectual disability and seizures among others. ASD is primarily genetic, with heritability estimates ranging from 83 to 91%^3–5^. Studies to date have identified hundreds of ASD genes, with causative *de novo* and inherited variants of differing effect sizes and frequencies, with approximately 100 of those genes presumed to harbor highly penetrant variants^6,7^. Additionally, risk-conferring copy number variants (CNVs) have been identified, highlighting the complex genetic architecture of ASD^8–15^. Each of the currently known ASD genes accounts for less than 1% of cases^7^, and all the rare variants identified to date account for only ~30% of the disease burden, in particular when considering families with complex phenotypes^16^.

Rare biallelic events are estimated to contribute to 5% of the ASD burden of disease, with that percentage increasing to 10% in females^17^. Variant discovery of rare alleles of large effect requires sequencing of large numbers of individuals, and for sufficiently rare alleles, it is unlikely that they will be observed in a homozygous state in a nonconsanguineous population. Previous studies have successfully analyzed runs of homozygosity in consanguineous families to discover rare variants contributing to recessive disease^18–22^. This has proved to be an effective strategy to identify genes that were previously not known to be involved in ASD^18,23–25^, contributing to the understanding of the underlying biology of the disorder.

In this study, we ascertained a familial cohort through probands with ASD and aimed to utilize the recent shared ancestry within the cohort to detect rare and ultra-rare pathogenic biallelic variants, which would be difficult to identify in nonconsanguineous cohorts. This unique collection of ancestrally diverse families had representation from the Middle East (47 individuals), South Asia (12 individuals), and Europe (5 individuals), in addition to one East Asian individual, one Hispanic individual, one individual of mixed European and East Asian ancestry, and one individual of mixed European and Hispanic ancestry. We performed whole genome sequencing (WGS) followed by homozygosity mapping and mining of regulatory element annotations, and focused on rare deleterious variants as candidate disease-causing. We identified 24 genes with 34 nonsynonymous exonic or splice site variants in 18 affected individuals. Of the identified genes, 12 have been previously implicated in ASD and other neurodevelopmental disorders and 12 are new candidate ASD genes expressed in the brain and characterized as having neurodevelopmental functions with potential consequences in disease. For 2 affected individuals, we identified CNVs overlapping with known ASD or neurodevelopmental disease loci. In addition, we identified 37 inherited homozygous variants within brain-specific regulatory elements, 5 of which were located within promoters or enhancers for known ASD genes. Overall, we identified potentially etiological variants in known ASD or neurodevelopmental disease genes for 14 out of 23 affected individuals. Biallelic events involving rare and ultra-rare variants seldom occur in nonconsanguineous populations. By leveraging the enriched homozygosity in this consanguineous cohort, we were able to identify biological processes and mechanisms that will generalize to ASD in other populations.

## Results

### Clinical characteristics of the ASD consanguineous cohort

A total of 22 families, including 20 trios, 1 quad with two affected siblings, and 1 quad with an affected proband and his unaffected fraternal twin, were enrolled in our study (see Supplementary Table 1). The majority of families (68%, 15/22) reported consanguinity through first-cousin unions. The cohort comprised a total of 23 affected individuals and their family members, with an affected male to female ratio of 6.7 (20 males, 3 females). Language and speech impairments were identified in all affected individuals tested (*N* = 16) and 56% of them were non-verbal. Other notable phenotypes included intellectual disability (5/9 affected individuals assessed), developmental delay (8/8), attention deficit hyperactivity disorder (4/8), and seizures (1/18) (see Table 1). All affected individuals tested had normal brain imaging on MRI and CT scans (*N* = 11).

**Table 1:** Demographics and clinical information for the consanguineous ASD cohort.

Clinical symptoms in probands	Probands assessed (*N*)	Probands with phenotype (*N*)
ASD	23	23
Speech impairment	16	16
* Verbal*		7
* Non-verbal*		9
Intellectual disability	9	5
Developmental delay	8	8
Seizures	18	1
Learning disabilities	8	4
Attention deficit hyperactivity disorder	8	4
Gastrointestinal problems	8	4

	Average (Range, years)
Age at diagnosis (*N* = 20)	2.7 (0.75–7.0)
Paternal age at birth (*N* = 10)	33.7 (24.3–45.7)
Maternal age at birth (*N* = 10)	31.0 (24.0–39.6)

The majority of families in our cohort (15/22) are of Jordanian descent, including 13 families recruited in Jordan and 2 families recruited in the United States (see Supplementary Table 1). We used principal component analysis (PCA) to explore the ancestry of the families in the cohort. The majority of samples in the cohort clustered adjacent to European samples from the 1000 Genomes project (1000G)^26^ with separation from other European subpopulations. In addition to individuals of Jordanian ancestry, our cohort includes individuals with ancestry from the Asian Subcontinent and a family from Peru. Samples from these families clustered as expected with the 1000G subpopulations from the corresponding regions (see Supplementary Fig. 1).

### Whole genome sequencing and variant discovery

We performed WGS on samples from 68 individuals including 23 affected children. The average read depth was 37X, with no differences in depth of sequencing with respect to affection status, sex, or family relationships (see Supplementary Figs. 2a–c). On average, 99.6% and 95.6% of bases were covered at a mean read depth of at least 10× and 20×, respectively (see Supplementary Fig. 2d).

An average of 4,819,156 total variants were identified per genome. After applying read depth and quality filters, 3,071,060 variants per genome remained, of which an average of 2,666,208 were single nucleotide variants (SNVs) and 404,852 were insertions or deletions (indels) (see Supplementary Table 2). A detailed summary of our WGS data processing and variant filtration pipeline is shown in Supplementary Fig. 3. We filtered for rare variants with a minor allele frequency (MAF) < 1% in all annotated population databases (1000G^26^, Genome Aggregation Database (gnomAD)^27^, and Greater Middle East Variome Project (GME)^28^), identifying on average 112,512 rare variants per genome, of which 110,450 were heterozygous and 2,063 were homozygous (see Supplementary Table 2). Affection status, sex, or family relationships had no significant effect on the average variant counts in any category (see Supplementary Fig. 4). We discovered an average of 34,840 ultra-rare variants per genome, and a total of 1,193,026 unique ultra-rare variants in the cohort that have not been reported in any of the public databases that we used for annotation (see Supplementary Table 2). Out of these variants an average of 5,580 were private (8567 for parents, 105 for offspring), meaning present only in a single individual in the cohort. We identified an average of 24 (36 for parents, 1 for offspring) private exonic or splice site (referred to as coding) variants per genome, of which 8 (13 for parents, 0.4 for offspring) per genome were nonsynonymous and predicted to be deleterious (see Supplementary Table 2).

To assess whether there was an excess of potentially pathogenic variants in affected compared to unaffected individuals, we performed a burden analysis. We found no difference between affected and unaffected individuals in the burden of rare variants with nondisrupting (ND), missense damaging (MD), or loss of function (LoF) effects (see Supplementary Fig. 5). This was expected due to the consanguinity in our cohort, the small sample size, and the lack of unaffected siblings (except for one family with one unaffected sibling).

### Analysis of copy number variation

We identified copy number aberrations in affected individuals by using CNVkit^29^. Briefly, average read depth in affected individuals was compared to the average read depth in unaffected family members across the genome. We identified an average of 310 genomic regions with copy number aberrations per affected individual, ranging in size from 2.2 Kb to 93.6 Mb. Out of the identified CNVs, a total of 1790 overlapped with known ASD CNVs reported in the CNV Module of the SFARI Gene database^6^, and were designated as “overlapping”. To further focus on potentially clinically relevant CNVs, we filtered for “overlapping” CNVs that were larger than the median size of the corresponding SFARI CNV in ASD cases, and we identified 22 such CNVs in 10 affected individuals (see Supplementary Table 3). To identify genomic regions that were significantly deleted or amplified across all affected individuals, we analyzed the output from CNVkit^29^ with GISTIC2.0^30^. We identified 113 significant regions (62 amplifications and 51 deletions), of which 19 (10 amplifications and 9 deletions) overlapped with known ASD CNVs. We then filtered for CNVs that were larger than the median size of the corresponding SFARI CNV, and we identified 4 such CNVs, 2 amplifications and 2 deletions (see Supplementary Table 4).

### Homozygosity analysis

Consanguineous populations have higher proportions of genomic homozygosity. Recent shared ancestry results in larger genomic blocks that are inherited identical by descent as compared to outbred populations^31^. Rare homozygous variants are therefore likely to be present in runs of homozygosity (ROH) in the offspring of consanguineous unions^32^. Due to the consanguinity in our cohort, we utilized homozygosity analysis to identify regions of the genome that are homozygous and likely to harbor rare recessive mutations.

**Fig. 1:**
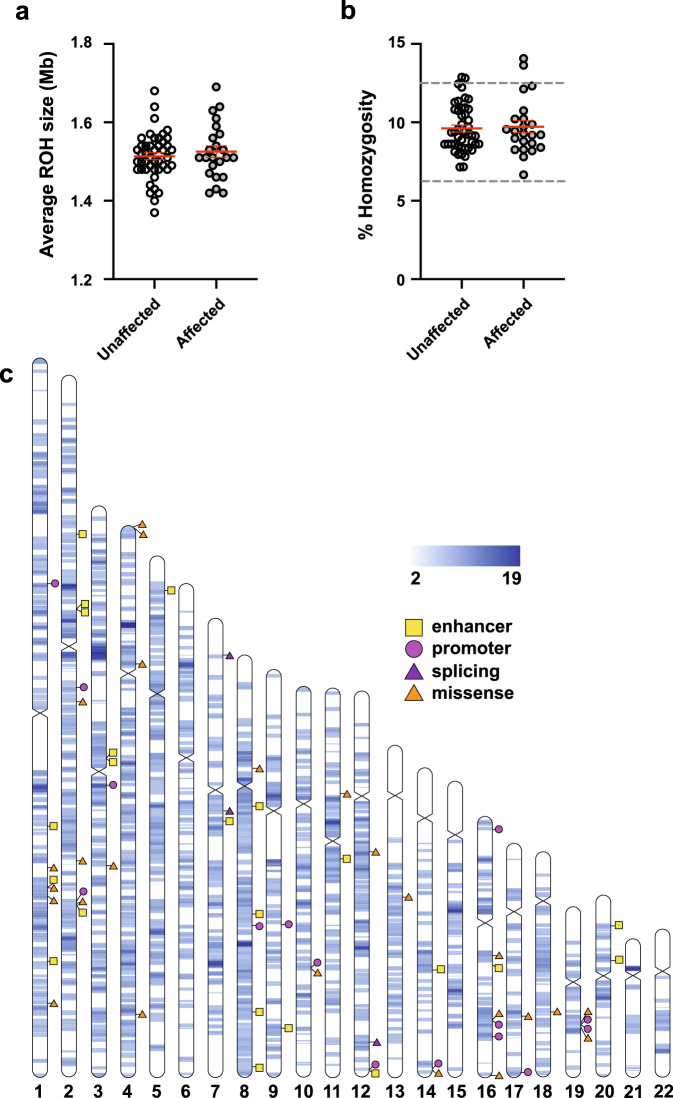
Runs of homozygosity in the consanguineous ASD cohort. There were no significant differences in average size of ROHs (**a**) or total percent of homozygosity across the genome (**b**) between affected and unaffected individuals (mean ± SEM are shown in red). In **b** dotted lines denote the expected % homozygosity for children of second- and third-degree relatives (12.5% and 6.25%, respectively). Data were analyzed using unpaired *t* test (*N* = 23 affected, 45 unaffected; *P* = 0.4599 (**a**), *P* = 0.7878 (**b**)). **c** ROHs shared between unrelated probands, with rare inherited homozygous nonsynonymous variants and brain-specific regulatory element variants that map within them. The color scale indicates the number of probands sharing an ROH.

We identified an average of 183 autosomal runs of homozygosity (ROHs) per genome that are each >1 Mb in size. The average ROH length was 1.52 Mb per genome (see Fig. 1a), with the largest ROH in each genome ranging from 3 Mb to 7.6 Mb. The total length of ROHs averaged 278.7 Mb (see Supplementary Fig. 6a) corresponding to 9.65% of the genome (see Fig. 1b). Since the expected homozygosity in genomes of offspring from first cousin and double-first cousin unions is 6.25% and 12.5%^31^, respectively, our results suggested a second to third degree relationship between parents in our cohort, in line with self-reported information from study participants. Apart from a minor increase in average ROH size in males, we saw no difference in ROH metrics in correlation with affection status, sex, or family relationships (see Fig. 1a and b, and Supplementary Fig. 6).

In order to identify shared ROH segments between affected individuals, we combined all ROHs that were found in at least 2 affected individuals to construct 448 merged ROH segments ranging in size from 1 Mb to 72.3 Mb (see Fig. 1c). Out of these merged ROHs, 31 ranging in size from 0.4 Mb to 5.8 Mb were shared in more than half of the affected individuals (see Fig. 1c and Supplementary Table 5). The two ROHs most commonly shared between affected individuals were a 4.7 Mb region on chromosome 3 (chr3: 48,187,679–52,913,780) and a 1.6 Mb region on chromosome 4 (chr4: 33,294,517–34,943,265). The chromosome 3 region spans 159 genes, including neuronal development genes *SEMA3F* and *SEMA3B*, and epilepsy-associated genes *NPRL2*, *CACNA2D2*, and *CYB561D2* (see Supplementary Fig. 7a). The chromosome 4 region contains three long non-coding RNAs (see Supplementary Fig. 7b).

**Fig. 2:**
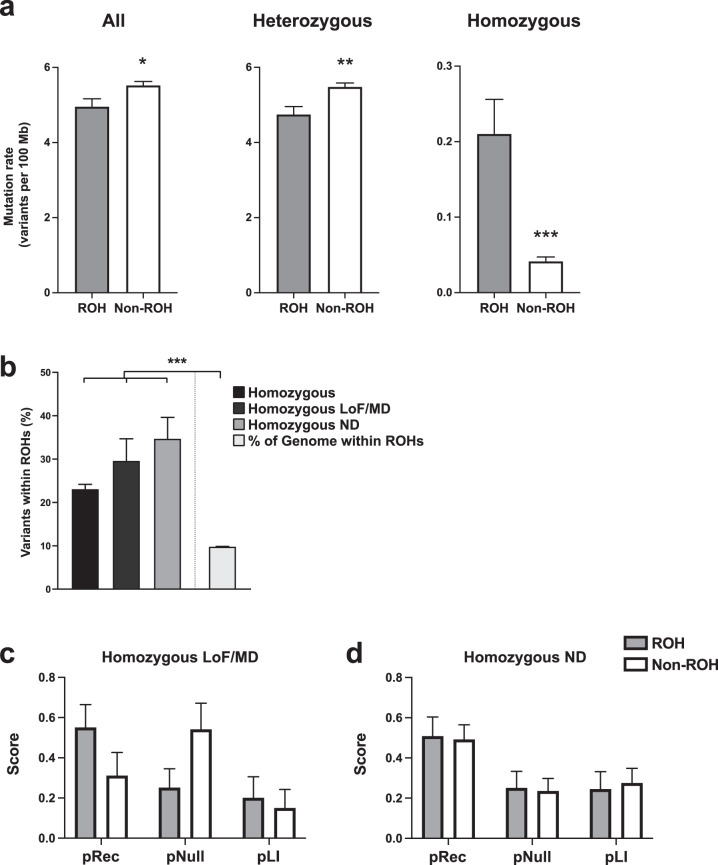
Damaging mutations are enriched within ROHs. **a** Rate of all rare (*P = 0.0176), rare heterozygous (**P = 0.0025), and rare homozygous (***P = 0.0004) LoF and MD variants per 10^8^ base pairs within and outside ROHs (*N* = 68 individuals). **b** The percentage of all, LoF/MD, and ND rare homozygous variants that fall within ROHs for each individual compared to the percentage of the genome that is within ROHs (***P < 0.0001 for each comparison). The percentage of rare homozygous variants within ROHs that are LoF/MD compared to ND were not different (*N* = 68 individuals; *P* = 0.4821). **c** Constraint scores for genes harboring rare inherited homozygous LoF/MD variants in affected individuals, represented by average pRec (*P* = 0.1543), pNull (*P* = 0.0818), and pLI (*P* = 0.7232) scores from gnomAD (*N* = 15 ROH genes, *N* = 14 non-ROH genes). **d** Constraint scores for genes harboring rare inherited homozygous ND variants in affected individuals, represented by average pRec (*P* = 0.8951), pNull (*P* = 0.8834), and pLI (*P* = 0.7884) scores from gnomAD (*N* = 24 ROH genes, *N* = 34 non-ROH genes). All values are mean ± SEM. Data were analyzed using unpaired *t* test.

We examined the burden of damaging mutations within ROHs by comparing the rate of rare LoF and MD variants within and outside ROHs for all individuals. We found that the rate of rare homozygous LoF and MD variants within ROHs were significantly higher than the rest of the genome (see Fig. 2a). We then examined the distribution of rare homozygous variation across the genome, and found that ROHs, which on average spanned 9.65% of the genome in our cohort, harbor 29.5% of rare homozygous LoF/MD variants (see Fig. 2b). This percentage did not differ significantly for rare homozygous ND variants within ROHs (34.6%) (see Fig. 2b), indicating that ROHs do not necessarily carry more damaging variation, but are rather enriched for all rare homozygous variation. Using data from gnomAD, we assessed the constraint of genes carrying rare inherited homozygous LoF and MD variants, and found that such genes within ROHs showed higher constraint and intolerance to homozygous LoF mutations (average pRec score = 0.5497, average pNull score = 0.2503, *N* = 15) compared to genes that carry the same category of variants but are outside ROHs (average pRec score = 0.3104, average pNull score = 0.5404, *N* = 14) (see Fig. 2c). Constraint for genes carrying rare inherited homozygous ND variants did not change whether the genes were within or outside ROHs (see Fig. 2d).

To identify candidate ASD variants within ROHs, we excluded all ROHs that were found in any of the parents. We found 4 ROH segments shared between two to four affected individuals, all smaller than 0.1 Mb (see Supplementary Table 6). There were no coding variants shared between the affected individuals carrying these regions, however, there were 76 shared noncoding variants (see Supplementary Table 7). To better interpret the functional impact of these variants, we annotated them using three publicly available ChIP-seq and ATAC-seq datasets: (1) chromatin state segmentation from nine human cell lines^33^, (2) maps of the activating histone modification H3K4me3 in human prefrontal cortex from 11 individuals^34^, and (3) predicted developmental brain enhancers from human fetal brain samples^35^. We identified an ROH on chromosome 3 (chr3: 18,335,384–18,359,238) carrying a shared variant that mapped to a predicted heterochromatic region. This ROH spans *SATB1*, which encodes an activity-dependent transcription factor that regulates neuronal development^36,37^, and *LOC339862*, a lncRNA gene found to be hypermethylated in senile plaques of postmortem human brains with Alzheimer’s disease^38^. Another shared ROH on chromosome 4 (chr4: 3,389,923–3,476,537) harbored several intronic variants that mapped onto predicted enhancers and transcriptional elongation/transition-related regions. The ROH spans *RGS12* which encodes a G-protein signaling regulator and transcriptional repressor with enhanced expression in the brain (based on data from the Genotype-Tissue Expression Portal, GTEx), and *DOK7*, which encodes a protein involved in neuromuscular junction formation, and shows moderate expression in the brain and enhanced expression in heart and skeletal muscle (GTEx). Another interesting shared ROH on chromosome 22 harbored a variant in a transcriptional elongation/transition-related region in an intron of *ZMAT5*, an RNA splicing/processing gene that is highly expressed in the brain (GTEx) (see Supplementary Table 7).

### Identification of candidate ASD variants

For candidate ASD variant discovery, we initially focused on rare exonic nonsynonymous or splice site variants in affected individuals that were either *de novo* or fit a recessive inheritance model. We identified an average of 80 *de novo* variants per proband (see Supplementary Table 8). This is slightly increased compared to previous reports of ~60–70 *de novo* variants per genome, however the average number of coding *de novo* variants was 1 per proband, similar to previous reports^39,40^. The lack of a public WGS dataset from a cohort of similar ancestry to our cohort, likely contributed to this moderate increase in the number of noncoding *de novo* variants. For coding *de novo* variants, on the other hand, we were able to filter against the GME, a whole exome sequencing dataset. In addition, we identified an average of 1251 inherited homozygous variants (6 coding) and 22 compound heterozygous variants in 10 genes per proband (see Supplementary Table 8). We also identified an average of 5 recessive X-linked variants in affected male offspring, none of which were coding (see Supplementary Table 8). We did not see a significant correlation between the number of *de novo* variants and maternal or paternal age at birth of an affected offspring which may be due to the small number of families for which parental ages were available (see Supplementary Fig. 8). In total, we identified 258 rare exonic nonsynonymous or splice site variants in 152 genes (see Supplementary Table 9). Out of these, 95 variants were in 42 OMIM-annotated disease genes associated with relevant phenotypes including intellectual disability, developmental delay, and epilepsy.

We cross-referenced the rare inherited homozygous variants within ROHs in affected individuals. We identified 63 rare homozygous coding variants (0–12 per genome) that were located within an ROH and were not present in homozygous form in the parents or an unaffected sibling (see Supplementary Table 8). Out of these, 39 variants (0–9 per genome) were nonsynonymous (see Fig. 1c and Supplementary Tables 8 and 9).

The majority of inherited homozygous variants within ROHs were noncoding. Annotation of these noncoding variants with the aforementioned ChIP-seq and ATAC-seq datasets identified an average of 3 (0–13) variants within predicted human brain promoters as well as an average of 2 (0–12) variants within predicted human brain enhancers per genome (see Supplementary Table 8). To identify regulatory variants that potentially underlie neurodevelopmental disease, we focused on brain-specific promoter and enhancer variants, of which there were 15 and 22, respectively (see Fig. 1c and Supplementary Table 10). A majority of the enhancer variants (20/22) were also associated with the activating chromatin markers H3K4me1 and H3K27ac (see Supplementary Table 10). We identified 49 inherited homozygous noncoding variants that were shared between two to three unrelated affected individuals. These shared variants showed weak to moderate associations with promoter, enhancer, and transcription elongation/transition regions based on the ENCODE dataset (see Supplementary Table 11). Two of these variants shared by two unrelated probands were located in the ASD-associated gene *EXOC6*.

We utilized multiple criteria to prioritize nonsynonymous coding and brain-specific noncoding variants as potentially causative for each affected individual. The prioritization or exclusion criteria for each variant are summarized in Supplementary Tables 9 and 10. Briefly, we considered (1) known association of the gene with ASD or other neurodevelopmental diseases, (2) gene expression in the brain from GTEx and Human Protein Atlas data^41^, (3) protein damage prediction and evolutionary conservation for coding variants, and (4) validation of the predicted promoter/enhancer interactions in a fourth human brain dataset for noncoding variants^42^. We identified 46 candidate variants in 36 genes or human brain-specific regulatory elements in 20 affected individuals (see Tables 2 and 3).

**Table 2:** Potentially pathogenic variants in known ASD and neurodevelopmental disease genes identified in affected individuals from the consanguineous ASD cohort.

Affected individual	Inheritance	Variant(s)	Variant type	Gene(s)	Variant location	Mutation(s)	Relevant OMIM phenotype(s)
JC-35-3	Compound Heterozygous	chr18:67,860,533:G>A; chr18:67,871,333:G>C	Missense	*RTTN*	Exon	p.T129S; p.A333V	Microcephaly, short stature, and polymicrogyria with seizures (AR)
JC-37-3	Homozygous	chr16:53,653,078:TA>T	Frameshift deletion	*RPGRIP1L*	Exon	p.L1112fs	Joubert syndrome (AR); Meckel syndrome (AR); COACH syndrome (AR)
JC-37-3	Homozygous (ROH)	chr16:76,312,429:CCCTT>C	Deletion	*CNTNAP4*	Promoter	─	─
JC-37-3	Homozygous (ROH)	chr17:79,475,862:C>T	SNV	*BAHCC1, ACTG1*	Promoter	─	Baraitser-Winter syndrome 2 (*ACTG1*)
JC-39-3	Homozygous (ROH)	chr5:11,828,012:A>C	SNV	*CTNND2*	Enhancer	─	─
JC-39-3	Homozygous (ROH)	chr7:70,146,191:G>A	SNV	*AUTS2*	Enhancer	─	Intellectual disability (AD)
JC-41-3	Homozygous (ROH)	chr16:89,753,128:C>G	Missense	*CDK10*	Exon	p.P4A	Al Kaissi syndrome (AR)
JC-50-3	Compound Heterozygous	chr7:151,884,538:G>A; chr7:151,896,483:T>C	Missense	*KMT2C*	Exon	p.P1606L; p.N1385S	Kleefstra syndrome 2 (AD)
JC-57-3	*De novo*	chr6:87,964,707:C>T	Stop gain	*ZNF292*	Exon	p.R454X	─
JC-58-3	Compound Heterozygous	chr4:183,714,156:C>T; chr4:183,713,475:G>A	Missense	*TENM3*	Exon	p.R2111W; p.D1884N	Syndromic microphthalmia (AR)
JC-58-3	Homozygous (ROH)	chr16:70,304,215:G>A	Missense	*AARS*	Exon	p.P234S	Charcot-Marie-Tooth disease (AD); Epileptic encephalopathy (AR)
JC-60-3	Compound Heterozygous	chr6:152,737,541:G>A; chr6:152,757,224:G>A	Missense	*SYNE1*	Exon	p.R2018C; p.R1395W	Emery-Dreifuss muscular dystrophy 4 (AD); Spinocerebellar ataxia 8 (AR)
JC-62-3	Compound Heterozygous	chr9:27,212,851:G>A; chr9:27,185,613:A>G; chr9:27,192,561:C>T	Missense	*TEK*	Exon	p.A902T; p.N395S; p.R479C	Primary congenital glaucoma 3E (AD); Venous malformations (AD)
JC-62-3	Homozygous (ROH)	chr19:40,972,808:G>A	SNV	*SPTBN4*	Promoter	─	Neurodevelopmental disorder with hypotonia, neuropathy, and deafness (AR)
MC-03-3	*De novo*	chr16:5,193,623–6,623,110	CNV - Deletion	*LOC100287538, RBFOX1, LOC440337*	16p13.3	─	─
MC-04-3	*De novo*	chr2:54,839,463:C>T	Missense	*SPTBN1*	Exon	p.R143C	Developmental delay, impaired speech, and behavioral abnormalities (AD)
MC-14-3	Homozygous	chr1:240,392,611–24,039,5129	CNV - Deletion	*FMN2*	1q43	─	Intellectual disability (AR)
MC-14-3	*De novo*	chr1:242,461,081–246,469,606	CNV - Duplication	*PLD5, LOC100505955, LOC731275, CEP170, MIR4677, SDCCAG8, AKT3, LOC339529, ZBTB18, LOC440742, C1orf100, ADSS2, C1orf101, DESI2, COX20, HNRNPU-AS1, HNRNPU, EFCAB2, KIF26B, SMYD3*	1q43-q44	─	Intellectual disability (AD); Developmental and epileptic encephalopathy 54 (AD); Megalencephaly-polymicrogyria-polydactyly-hydrocephalus syndrome 2 (AD)
MC-17-3	*De novo*	chr2:166,848,378:CCTCA>C	Frameshift deletion	*SCN1A*	Exon	p.S1801fs	Dravet syndrome (AD); Epilepsy (AD)
MC-32-3	Compound Heterozygous	chr11:6,652,592:G>A; chr11:6,654,195:G>A	Missense	*DCHS1*	Exon	p.A1241V; p.R850C	Van Maldergem syndrome 1 (AR)

List of deleterious coding, brain-specific regulatory noncoding, and copy number variants affecting known ASD or neurodevelopmental disease genes identified for each affected individual. ROH indicates inherited homozygous variants that are within runs of homozygosity. For SFARI score, S denotes syndromic genes.

*AD* autosomal dominant, *AR* autosomal recessive.

**Table 3:** Candidate variants in putative ASD genes identified in affected individuals from the consanguineous ASD cohort.

Affected individual	Inheritance	Variant(s)	Variant type	Gene(s)	Variant location	Mutation(s)	pLI score	LOEUF score
JC-37-3	Homozygous	chr8:95,802,020:G>T	Missense	*DPY19L4*	Exon	p.R685L	0	1.07
JC-38-3	Compound Heterozygous	chr12:110,221,470:G>A; chr12:110,234,488:G>A	Stop gain; missense	*TRPV4*	Exon	p.Q751X; p.R392W	0	1.06
JC-39-3	*De novo*	chr11:46,679,092:AC>A	Frameshift deletion	*ATG13*	Exon	p.P206fs	0.96	0.33
JC-39-3	Homozygous	chr2:196,659,081:A>G	Missense	*DNAH7*	Exon	p.F3566S	0	0.84
JC-39-3	Homozygous (ROH)	chr14:104,312,220:G>T	SNV	*PPP1R13B*	Promoter	─	0.998	0.27
JC-40-3	*De novo*	chr7:50,685,818:T>C	Missense	*GRB10*	Exon	p.M260V	0.94	0.34
JC-50-3	Homozygous (ROH)	chr16:4,234,820:T> TCACCTTGGCTGCTCTTCCATTCCCTTATCCTCGCCACA	Insertion	*SRL, ADCY9*	Enhancer	─	0; 0.98	1.21; 0.31
JC-56-3	Compound Heterozygous	chr22:38,221,032:C>T; chr22:38,220,770:C>A	Missense	*GALR3*	Exon	p.A221V; p.R134S	0	1.71
JC-62-3	Homozygous (ROH)	chr8:93,075,094:C>T	SNV	*RUNX1T1*	UTR5, Promoter	─	0.978	0.31
MC-03-3	*De novo*	chr1:6,202,478:T>C	Missense	*CHD5*	Exon	p.K744R	1	0.16
MC-12-3	Compound Heterozygous	chr12:132,629,445:G>A; chr12:132,633,428:G>A	Missense	*NOC4L*	Exon	p.R55H; p.A297T	0	0.62
MC-12-3	Homozygous (ROH)	chr7:66,459,197:A>G	Splicing	*SBDS*	Splice site		0	1.23
MC-14-3	Homozygous (ROH)	chr8:38,947,571:A>T	Missense	*ADAM9*	Exon	p.N692Y	0.03	0.42
MC-14-3	Homozygous	chr11:32,976,929:G>T	Missense	*QSER1*	Exon	p.G1401C	1	0.21
MC-14-3	Homozygous (ROH)	chr1:183,522,140:G>A	Missense	*SMG7*	Exon	p.G1114E	1	0.22
MC-14-3	Homozygous (ROH)	chr1:183,263,559:G>A	SNV	*SMG7, NMNAT2*	Enhancer	─	1; 0.99	0.22; 0.27
MC-24-3; MC-24-4	Homozygous (ROH)	chr1:77,749,698:T>G	SNV	*AK5*	Promoter	─	0	0.56
MC-24-3; MC-24-4	Homozygous (ROH)	chr8:142,639,754:T>C	SNV	*TSNARE1, ADGRB1*	Enhancer	─	0; 1	0.83; 0.14

List of high-priority deleterious coding and brain-specific regulatory noncoding variants in putative ASD genes identified for each affected individual. Coding variants were prioritized based on deleteriousness and effect on conserved residues, and genes expressed highly in the human brain. Noncoding variants were prioritized based on overlap with brain-specific promoter and/or enhancer regions. ROH indicates inherited homozygous variants that are within runs of homozygosity.

*AD* autosomal dominant, *AR* autosomal recessive.

#### Variants in known ASD or neurodevelopmental disease genes

Table 2 summarizes the potentially pathogenic variants in known ASD or neurodevelopmental disease genes for each affected individual. We identified 27 variants in 14 affected individuals (~1–3 per individual), including coding variants in 12 genes, variants in 5 brain-specific regulatory regions (3 in promoters, 2 in enhancers), and 3 CNVs.

Five affected individuals had coding variants in syndromic ASD genes: JC-50-3 in *KMT2C*, JC-57-3 in *ZNF292*, JC-60-3 in *SYNE1*, MC-04-3 in *SPTBN1*, and MC-17-3 in *SCN1A*. Proband MC-04-3 presented with ASD, speech abnormalities, and developmental delay, in line with phenotypes of patients with pathogenic *SPTBN1* mutations (MIM #619475)^43,44^. In addition to ASD, proband MC-17-3 presented with epilepsy, the characteristic phenotype in patients with *SCN1A* loss of function mutations. Five other probands (JC-35-3, JC-37-3, JC-41-3, JC-58-3, and MC-32-3) had coding variants in neurodevelopmental disease genes: *RTTN* (MIM #614833)^45^, *RPGRIP1L* (MIM #619113, #611560, #611561)^46–48^, *CDK10* (MIM # 617694)^49^, *AARS* (MIM #613287, #616339)^50,51^, *TENM3* (MIM #615145)^52^, and *DCHS1* (MIM #601390)^53–55^. In addition to ASD, individual JC-35-3 presented with intellectual disability and lack of speech, both of which are phenotypes associated with recessive loss of function mutations in *RTTN*^45,56,57^. *RPGRIP1L* mutations are known to cause Joubert (MIM #213300)^48^, Meckel (MIM #611561), and COACH (MIM #216360)^46^ syndromes, characterized by intellectual disability and gross brain defects. *TENM3* encodes a transmembrane protein involved in the regulation of neuronal development^58^. Recessive *TENM3* mutations have been identified in syndromic microphthalmia with developmental delay and speech abnormalities, and some cases presenting with intellectual disability^52^. CNVs and noncoding variants in this locus have been previously associated with ASD^59–61^. Recessive mutations in *DCHS1* result in Van Maldergem syndrome (MIM #601390)^53,54^. Patients with Van Maldergem syndrome have been reported to have ASD, lack of speech, and developmental delay^55^, all of which are phenotypes of proband MC-32-3. For proband MC-14-3, our CNV analysis identified one small deletion within 1q43 and one duplication spanning the 1q43-1q44 boundary. Microdeletions and microduplications within 1q43-q44 are associated with neurodevelopmental phenotypes including intellectual disability, developmental delay, and limited or no speech^62,63^ (MIM #612337), all of which were present in the proband. Furthermore, both of these CNVs overlapped with SFARI-annotated ASD genes: the deletion is located within an intron of *FMN2* and the duplication spans *HNRNPU* and *ADSS2*.

In addition to coding variants, we identified noncoding variants in three affected individuals that mapped to 5 loci predicted to be brain-specific regulatory regions for known ASD and neurodevelopmental disease genes (see Table 2). These noncoding variants were within promotor or enhancer elements for the ASD genes *AUTS2*, *CNTNAP4*, and *CTNND2*, as well as neurodevelopmental disease genes *ACTG1* (MIM #614583)^64^ and *SPTBN4* (MIM #617519)^65^.

#### Variants in new candidate ASD genes

We identified 15 potentially pathogenic coding variants in 12 candidate ASD genes (see Table 3). Several of the candidate ASD genes that we identified encode proteins that function in neuronal development and connectivity (e.g., *CHD5*, *GRB10*). CHD5 is a chromatin remodeler that regulates neuronal differentiation and cortical development^66^, and its disruption in mice results in ASD-like behaviors^67^. A recent report identified heterozygous missense and LoF mutations in *CHD5* in an autosomal dominant neurodevelopmental disorder of intellectual disability, developmental delay, language deficits, and epilepsy^68^. GRB10 is involved in neuronal development and social dominance behavior in mice, and has a tissue-specific and imprinted expression pattern where the paternal allele is exclusively expressed in neurons^69^. GRB10 is also a known interactor of GIGYF1, encoded by a high-confidence ASD risk gene^70^. Mutations in the cation-encoding gene *TRPV4* are associated with motor neuropathy and sensory abnormalities^71^.

We identified 6 noncoding (3 promoter, 3 enhancer) candidate ASD variants that are located within human brain-specific regulatory regions (see Table 3). These variants are located in regulatory regions for neuropsychiatric and neurodegenerative disease genes, including *TSNARE1*^72^ and *ADCY9*^73^. Other genes predicted to be affected by a noncoding variant include *SMG7*, involved in nonsense-mediated mRNA decay regulation, a pathway that has been previously implicated in ASD and related disorders^74,75^. Differential methylation of the *SMG7* and *SMG7*-*AS1* promoters was associated with *SMG7* overexpression in an ASD patient^76^. Interestingly, proband MC-14-3 carries a deleterious coding variant within *SMG7* as well as the enhancer variant. Another interesting variant was identified in affected siblings MC-24-3 and MC-24-4, in an enhancer region that interacts with the promoters of *TSNARE1*, a gene associated with schizophrenia, and *ADGRB1*, a critical regulator of spine and synapse development^77^. Conserved transcription factor binding site variants in *ADGRB1* have also been previously identified in ASD patients^8^.

## Discussion

We performed WGS in a consanguineous cohort consisting of 68 individuals in 22 families, with at least one child in each family diagnosed with ASD. We used homozygosity mapping to identify runs of homozygosity and analyzed rare alleles in these segments. The largest autosomal ROH identified was a 7.6 Mb segment found in an affected individual. The average fraction of the genome under ROHs was 9.65%, larger than the expected percentage of shared homozygosity for children of first cousins (6.25%)^78^. The size of the larger blocks of homozygosity we observed are consistent with observations in other consanguineous cohorts and are indicative of previous generations of consanguinity^78^. Analysis of shared ROHs between affected individuals in our cohort showed no common genomic region harboring potentially causative variants. We also did not identify any deleterious coding, human brain-specific noncoding, or copy number variation within ROHs that were shared across unrelated probands. This once again highlights the genetic heterogeneity of ASD, and indicates that for ASD, analysis of ROHs is more informative when done on a within-family basis, to help identify potentially causative variants in each affected individual. Burden analysis showed that rare inherited homozygous LoF and MD variants are enriched in ROHs, and that the genes harboring these mutations are less likely to tolerate such mutations (see Fig. 2). This supports what is known about consanguinity in that it increases the risk of recessive disease in the offspring, and demonstrates that homozygosity mapping is an effective method to identify genomic regions that likely harbor recessive disease-causing mutations. We analyzed ROHs absent from parents and unaffected siblings and shared between unrelated probands, and we identified rare homozygous variants within these regions that were also shared between the probands. We identified 4 genomic regions each harboring at least one brain-expressed gene, as well as rare homozygous variants within these genes that were shared between affected individuals carrying each ROH. While homozygous events are enriched in consanguineous cohorts, compound heterozygous and *de novo* events are also expected. Our analysis identified 34 potentially pathogenic rare coding variants in 24 genes (see Tables 2 and 3). Eighteen of the 24 candidate genes carried biallelic events, and for 5 of these genes, these were located within an ROH. In addition, we identified 37 rare homozygous ROH variants that were located within brain-specific regulatory elements, 5 of which were located within or near a known ASD gene (see Supplementary Table 10). We also identified CNVs that overlap with known ASD CNV regions in 10 affected individuals (see Supplementary Table 3).

Tables 2 and 3 summarize the variants we identified in known ASD and neurodevelopmental disease genes and in new candidate ASD genes in each affected individual, respectively. In each of 12 affected individuals, we identified a single exonic variant (or 2 variants in case of compound heterozygosity) segregating with phenotype and meeting our criteria for deleteriousness, making these variants putatively pathogenic and the respective genes potentially causative. Five of these genes are known ASD genes (*KMT2C*, *SCN1A*, *SPTBN1*, *SYNE1*, *ZNF292*), 3 underlie neurodevelopmental disease but have not been previously implicated in ASD (*CDK10*, *DCHS1*, *RTTN*), and 4 are new candidate ASD genes (*CHD5*, *GALR3*, *GRB10*, *TRPV4*). For three probands, no candidate ASD variants were identified, and for the remaining 8 affected individuals, we identified ~2–6 candidate ASD variants, including exonic *de novo* or biallelic variants and homozygous variants in human brain-specific regulatory regions. In these cases, with multiple rare potentially pathogenic variants including those in ROHs, further functional investigation is needed to determine the potential causality of the identified variants.

Although consanguineous cohorts are rich for biallelic events, *de novo* variation is still present and is expected to contribute to disease burden. Five probands each carried 1 *de novo* candidate variant and no candidate biallelic events. MC-04-3 carries a *de novo* missense variant in the ASD gene *SPTBN1*^43,44^. Proband MC-17-3 carries a *de novo* frameshift deletion in *SCN1A*, in line with his epilepsy phenotype, and proband JC-57-3 carries a *de novo* nonsense variant in the syndromic ASD gene *ZNF292*. This mutation (p.R454X) has previously been reported in a patient with mild developmental and speech delays but not with ASD^79^. For each of JC-40-3 and MC-03-3, single candidate genes with no prior implication in any neurodevelopmental disease were identified, suggesting *GRB10* and *CHD5* as putative ASD genes. Two additional putative ASD genes were identified in probands carrying compound heterozygous variants: JC-38-3 in *TRPV4* and JC-56-3 in *GALR3*.

By utilizing WGS and homozygosity mapping, we were able to identify candidate biallelic variants within human brain-specific regulatory regions for known ASD and neurodevelopmental disease genes as well as new candidate ASD genes. In family MC-24, the two affected siblings shared a candidate ASD variant in an enhancer element linked to *TSNARE1* and *ADGRB1*, two genes with known neuronal functions^77,80,81^, as well as associations to ASD^8^, schizophrenia^72^, and Parkinson’s disease^82^. Another affected individual, JC-39-3, carried homozygous variants in brain-specific enhancers for 2 known ASD genes, *AUTS2* and *CTNND2*. Identification of additional regulatory element variants and further characterization of their functional impact will contribute to our understanding of ASD etiology and the landscape of ASD genetics.

In total, our analysis revealed potentially etiological ASD variants in 14 out of 23 affected individuals. We provide further evidence of the contribution of biallelic events to ASD and of the importance of analyzing genomic data from consanguineous cohorts to identify rare recessive coding variants, as well as evaluating the potential contribution of noncoding variants. This approach is an effective means of discovering genes underlying ASD, promoting further investigation and understanding of the biological underpinnings of disease.

## Methods

### Subjects and specimens

All human studies were reviewed and approved by the institutional review board (IRB) of the University of Texas Southwestern Medical Center (UTSW), the research committee at the University of Jordan School of Medicine, the ethics committee of the Jordan University Hospital, and the IRB of the Jordan University of Science and Technology. Families were recruited either from Jordan or from the Dallas Fort Worth area and written informed consent was obtained from all study participants. Inclusion criteria included a diagnosis of autism spectrum disorder (ASD) by a neurologist, child psychiatrist, or psychologist. Patients with genetically defined syndromes, specifically Fragile X syndrome, Angelman syndrome, Rett syndrome, or Tuberous sclerosis complex, were excluded from study participation. All patients enrolled in the study received a diagnosis of ASD from their referring clinicians who performed physical and behavioral assessments and administered the standard autism diagnostic measures (ADOS, ADI-R, and DSM-V). Blood samples were collected from all available family members by peripheral venipuncture and genomic DNA was isolated from circulating leukocytes using AutoPure (Qiagen, Hilden, Germany) according to the manufacturer’s instructions.

### Whole genome sequencing and data processing

Sequencing was performed in collaboration with the Autism Speaks MSSNG Consortium^83^ and was carried out at The Centre for Applied Genomics at The Hospital for Sick Children (Toronto, Canada) on the Illumina HiSeq X platform as previously described^83–85^. Briefly, DNA quality and quantity were assessed using a Qubit High Sensitivity Assay. Between 100 ng and 1 μg of DNA was used for genomic library preparation using the Illumina TruSeq Nano DNA Library Prep Kit according to the manufacturer’s protocol and libraries were paired-end sequenced (150 bp read lengths)^86^.

The genomes were processed as previously described^83^ following the best practices recommended by the Broad Institute^87^. Reads were aligned to the human reference genome version GRCh37/hg19 using the Burrows-Wheeler Aligner (BWA, version 0.7.10). Duplicate reads were removed using Picard (version 1.117). Local realignment and quality recalibration were performed using the Genome Analysis Toolkit (GATK; version 3.3). Variants (single nucleotide variants (SNVs) and insertions or deletions (indels)) were detected using GATK with HaplotypeCaller. Quality control checks for (i) duplicate samples, (ii) samples per platform, (iii) genome call rate, (iv) missingness rate, (v) singleton rate, (vi) heterozygosity rate, (vii) homozygosity rate, (viii) Ti/Tv ratio, (ix) inbreeding coefficient, and (x) sex inference were performed as previously described^83^. Variant call format (VCF) files for SNVs and indels were annotated with ANNOVAR using allele frequencies from the 1000 Genomes project (2015; 1000 G)^26^, the Genome Aggregation Database (gnomAD)^27^, and the Greater Middle East Variome Project (GME)^28^. Annotated VCF files were uploaded into a SQL database for working storage and analysis. Genome data was stored and analyses were performed on the Texas Advanced Computing Center (TACC) high-performance computing servers, a resource of the University of Texas (Austin, TX).

### Variant filtration

Variants were quality filtered in SQL using the PASS filter, a genotype quality (GQ) score of ≥ 99, and allelic read depth of ≥10. Rare variants were defined as those with minor allele frequencies (MAF) < 1% in 1000 G, gnomAD, and GME. Ultra-rare variants were identified by filtering for MAF = 0 in 1000 G, gnomAD, and GME. Private variants were defined as ultra-rare variants that occurred only in a single individual in our cohort. *De novo* variants were defined as any variant not present in the genome of either the father, the mother, or the sibling of a proband when available. To minimize potential false positive *de novo* calls, we applied additional filtering steps, requiring that *de novo* variants have the following criteria: (i) the ratio of sequence reads supporting the alternative call to total calls is between 0.3–0.7 for all variants in female probands and all autosomal and pseudoautosomal variants in male probands, and ≥ 0.7 for variants on the X and Y chromosomes in male probands, (ii) QD ≥4 and ReadPosRankSum ≥ −2.5^39^, (iii) the variant does not overlap with any rare variant in any other individual in our cohort, (iv) variant MAF < 0.1% in 1000G, gnomAD, and GME, (v) variant size of <50 bp for indels, (vi) the variant does not fall within known segmental duplications or simple repeat regions. To identify compound heterozygous variants in affected individuals, we selected rare coding (exonic or splicing) heterozygous events that were present in heterozygous form in one parent but not the other, then filtered for inherited variants within the same gene. Homozygous inherited variants were required to be present in heterozygous form in both the father and the mother, excluding variants that are homozygous in one of the parents on the assumption of full penetrance. X-linked variants were present in a male offspring and heterozygous in the mother. We also excluded pseudoautosomal variants that were heterozygous in the male offspring, and X-linked variants present in the unaffected father.

### Noncoding variant annotation

Custom SQL and Python scripts were used to annotate noncoding variants using three datasets: (1) chromatin state segmentation from nine human cell lines^33^, (2) maps of histone H3K4me3 mark in human prefrontal cortex (PFC) from 11 individuals^34^, and (3) predicted developmental brain enhancers from fetal brain samples^35^. The columns in supplementary tables derived from each dataset were denoted as ENCODE, uMass, and CBA, respectively. Additional details are presented in Supplementary Table 12. Variants that were found within a peak in the uMass dataset were marked as “predicted human brain promoter” variants. Predicted human brain promoter variants that were absent from regions with “1_Active_Promoter” prediction in any one of the 9 non-neuronal cell lines in the ENCODE dataset were marked as “predicted human brain-specific promoter” variants. Variants that were found within a predicted regulatory element (pRE) region in the CBA dataset were marked as “predicted human brain enhancer” variants. Predicted human brain enhancer variants that were absent from regions with “4_Strong_Enhancer” or “5_Strong_Enhancer” prediction in any of the 9 non-neuronal cell lines in the ENCODE dataset were marked as “predicted human brain-specific enhancer” variants. To verify regulatory element prediction, brain-specific enhancer and promoter variants were visualized using a UCSC genome browser track of brain cell-type specific proximity ligation-assisted ChIP-seq (PLAC-seq) data from Nott et al.^42^. PLAC-seq identifies long-range chromatin interactions at promoters and enhancers. We marked variants as linked to a certain gene if the enhancer region where the variant is located (based on the ChIP-seq and ATAC-seq data from the aforementioned UCSC genome browser tracks) was linked to the promoter of the target gene in the PLAC-seq data.

### Variant prioritization

For individual JC-35-3, compound heterozygous and homozygous inherited variants that were present in unaffected sibling JC-35-5 were excluded from analysis. For MC-24-3 and MC-24-4, compound heterozygous and homozygous inherited variants that were not shared by the affected siblings were excluded. *De novo*, compound heterozygous, inherited homozygous, and X-linked variants that are rare were considered to be potentially pathogenic if they met the following criteria: exonic or splice site, with an effect on the protein resulting in either a frameshift indel, a stopgain or stoploss, or nonsynonymous. We also kept variants with “unknown effect” to retain splice site variants in our analysis. For missense variants, we considered those that met at least two of the following criteria, with specific score cutoffs based on the cited published literature: SIFT score <0.05^88^, PolyPhen-2 HumVar score >0.15^89^, PROVEAN score < −2.5^90^, and MutationAssessor score >2.26^91^. PolyPhen-2 HumVar was chosen over PolyPhen-2 HumDiv because the former is more appropriate for Mendelian variants with drastic effect as we expect for ASD, while the latter is appropriate for common variants of smaller effect size. To assess evolutionary conservation of residues with missense variants, we considered CADD^92^, phastCons, phyloP^93^, and GERP++ ^94^ scores. Gene constraint was assessed using gnomAD pLI and Z scores^27^. We also inspected conservation across species by identifying gene homology groups using NCBI HomoloGene, and visualizing in Geneious Prime using Clustal Omega alignments^95^. The filtered variants were compared to the list of genes implicated in ASD from the Simons Foundation Autism Research Initiative (SFARI) Gene 2018 database^6^ (using the latest version updated in February 2020) and a list of established neurodevelopmental disease genes^7^. Variants were also screened for any phenotypic association in the Online Mendelian Inheritance in Man (OMIM) database. As an additional prioritization step, we deprioritized coding variants with MAF ≥1% and noncoding variants with a MAF >0.1%, or incidences of any homozygous alleles, in each subpopulation of 1000G and gnomAD. We especially focused on subpopulations relevant to the ancestry of each family in our cohort (i.e. SAS for MC-03, MC-17, MC-21, MC-32; EUR and EAS for MC-12; EUR and ASJ for MC-16; EUR for MC-04 and MC-24; and GME Syrian desert subpopulation for all Jordanian families).

Each potentially pathogenic coding or brain-specific noncoding variant was considered as high, medium, or low priority based on the criteria above. Priority rankings and the specific ranking criteria are included in Supplementary Tables 9 and 10. We selected high priority variants for each affected individual as the potentially pathogenic candidate ASD variants (Tables 2 and 3). If an affected individual did not carry any variants that were high priority, we selected medium priority variants.

### Copy number variant (CNV) analysis

We used CNVkit^29^ to detect CNVs based on read depth in affected samples relative to the average read depth in unaffected samples in the same family as controls, following general protocols^96^. We used GISTIC2.0^30^ on segmented files generated from CNVkit^29^ to further evaluate the significance of the amplified and deleted segments between the affected and unaffected samples. The criteria included a threshold for copy number amplification and deletion of 0.1, confidence level of 99 %, and FDR of 0.05. Proband CNVs that overlap with SFARI-annotated ASD CNVs were identified using R Bioconductor package regioneR^97^. Significance of the overlaps was tested by performing an overlap permutation test, also using regioneR^97^.

### Burden analysis

Nondisrupting (ND) variants were defined as exonic synonymous SNVs or exonic non-frameshift indels. Missense damaging (MD) variants were defined as exonic nonsynonymous SNVs that met at least two of the following criteria: SIFT score <0.05, PolyPhen-2 HumVar score >0.15, PROVEAN score < −2.5, and MutationAssessor score >2.26. Loss of function (LoF) variants were defined as splice site variants, and exonic variants predicted to result in a stopgain, stoploss, or frameshift indel. For the total burden analysis, the numbers of rare variants in each category (ND, MD, LoF) were compared between affected and unaffected individuals. For the analysis of burden within ROHs, mutation rates for ND and for LoF/MD variants were calculated by dividing the number of variants by the total length of ROHs for each individual. For the analysis of gene constraint, average pRec, pNull, and pLI scores from gnomAD were compared for genes carrying rare inherited homozygous ND or LoF/MD variants that are within or outside of a ROH in the affected individual who carries the variant. Briefly, pRec, pNull, and pLI describe the probability that a particular gene is recessive, unconstrained, or LoF-intolerant (likely haploinsufficient), respectively^27^. Genes with higher pRec and pLI scores are more likely to be intolerant to homozygous and heterozygous LoF variants, respectively. Genes with higher pNull scores are more likely to be completely tolerant of LoF variation.

### Assessment of runs of homozygosity

PLINK version 1.90b6.7^98,99^ was used for all analyses. VCF files were converted into PLINK format using vcftools version 0.1.13. The cohort was assessed for relatedness using PLINK-genome. Variants were filtered for Hardy-Weinberg equilibrium (*P* < 0.001), MAF > 5%, and maximum missing genotype rate of 25%. Runs of homozygosity (ROHs) were identified in PLINK using a sliding window analysis with a 100 base pair window size, allowing for 30 heterozygous variants and 30 missing genotypes per window in accordance with previously described methods^18^. The resulting segments were then assessed using the percent homozygous (PHOM) output from PLINK using thresholds of 50%, 70%, 75%, and 80%. The homozygosity threshold of 75% yielded genome-wide homozygosity in ROHs that were in accordance with expectations of homozygosity from relatedness determined through PLINK-genome and this threshold was used to filter ROHs for further analysis. Per-genome ROH metrics were calculated using autosomal ROHs only. Percentage of genome within ROHs was estimated as the ratio of total ROH length to total bases sequenced at 1×. Custom Python scripts were used to identify ROHs that were present in affected individuals and absent from their respective parents, and to identify the boundaries and the counts for ROHs that were shared between affected individuals. R package RIdeogram was used to visualize shared ROHs^100^.

### Variant validation

Candidate variants selected for validation were either in runs of homozygosity, or were *de novo* heterozygous or compound heterozygous variants in genes from the SFARI Gene 2018 database or from a list of neurodevelopmental disease genes^7^. A total of 61 candidate variants were assessed by targeted Sanger sequencing and 60 were validated (Supplementary Table 13). Genomic sequence surrounding the variant was downloaded from the University of California, Santa Cruz (UCSC) genome browser version GRCh37/hg19. PCR primers were designed to isolate and amplify the region surrounding a variant using the NCBI Primer-BLAST. Sequencing primers were designed using Primer3 version 0.4.0. PCR was carried out using standard protocols. Sequencing was performed at Genewiz (South Plainfield, NJ) or at the Eugene McDermott Center for Human Growth and Development Center Sequencing Core Facility at UTSW (Dallas, TX). The list of validated variants and sequences of the primers used can be found in Supplementary Table 13.

### Principal component analysis

Principal component analysis (PCA) was carried out in PLINK version 1.90b6.7^98,99^ using Phase 3 1000G data. PCA input files from our samples were created from VCF files using vcftools version 1.13 and were pruned to remove variants with MAF < 5%, missing genotype rate greater than 5%, and pruned for linkage disequilibrium (LD) with an r^2^ threshold of 0.2 using PLINK -indep-pairwise 50 5 0.2. Triallelic and palindromic variants were also removed. The set of variants that remained was extracted from the 1000 G dataset and these were merged with our cohort dataset. PCA was run in PLINK using the -pca flag and the first two principal components were plotted in R.

### Web resources

1000G: https://www.internationalgenome.org/data; Allen Brain Atlas: http://www.brain-map.org; gnomAD Browser: http://gnomad.broadinstitute.org; GME: http://igm.ucsd.edu/gme; GTEx Portal: https://www.gtexportal.org/home; NCBI Primer-BLAST: https://www.ncbi.nlm.nih.gov/tools/primer-blast/; OMIM: http://www.omim.org; PLINK: http://pngu.mgh.harvard.edu/purcell/plink/; Primer3: http://bioinfo.ut.ee/primer3-0.4.0/; UCSC Genome Browser: http://genome.ucsc.edu.

### Reporting summary

Further information on research design is available in the Nature Research Reporting Summary linked to this article.

## Supplementary information

Supplementary Figures

Supplementary Tables

Reporting summary

## Data Availability

Sequence data can be accessed through the Autism Speaks MSSNG database (for access, see https://research.mss.ng/). MSSNG is a well-established whole genome sequence resource utilized by approved investigators worldwide. Sequence data have also been deposited at the European Genome-phenome Archive (EGA), which is hosted by the EBI and the CRG, under accession number EGAS00001005938.

## Code availability

Codes used in the MSSNG database have been published^83^ and can be accessed here: https://www.ncbi.nlm.nih.gov/pmc/articles/PMC5501701/. Others are available upon request.
