# Supplementary material for: Analysis of recent shared ancestry in a familial cohort identifies coding and noncoding autism spectrum disorder variants
Source: NPJ Genom Med. 2022 Feb 21;7:13. doi: 10.1038/s41525-022-00284-2 (PMC8861044; doi:10.1038/s41525-022-00284-2)
Supplement: Supplementary file 1 — Supplementary Figures [file 41525_2022_284_MOESM1_ESM.pdf]

Supplementary Figure 1

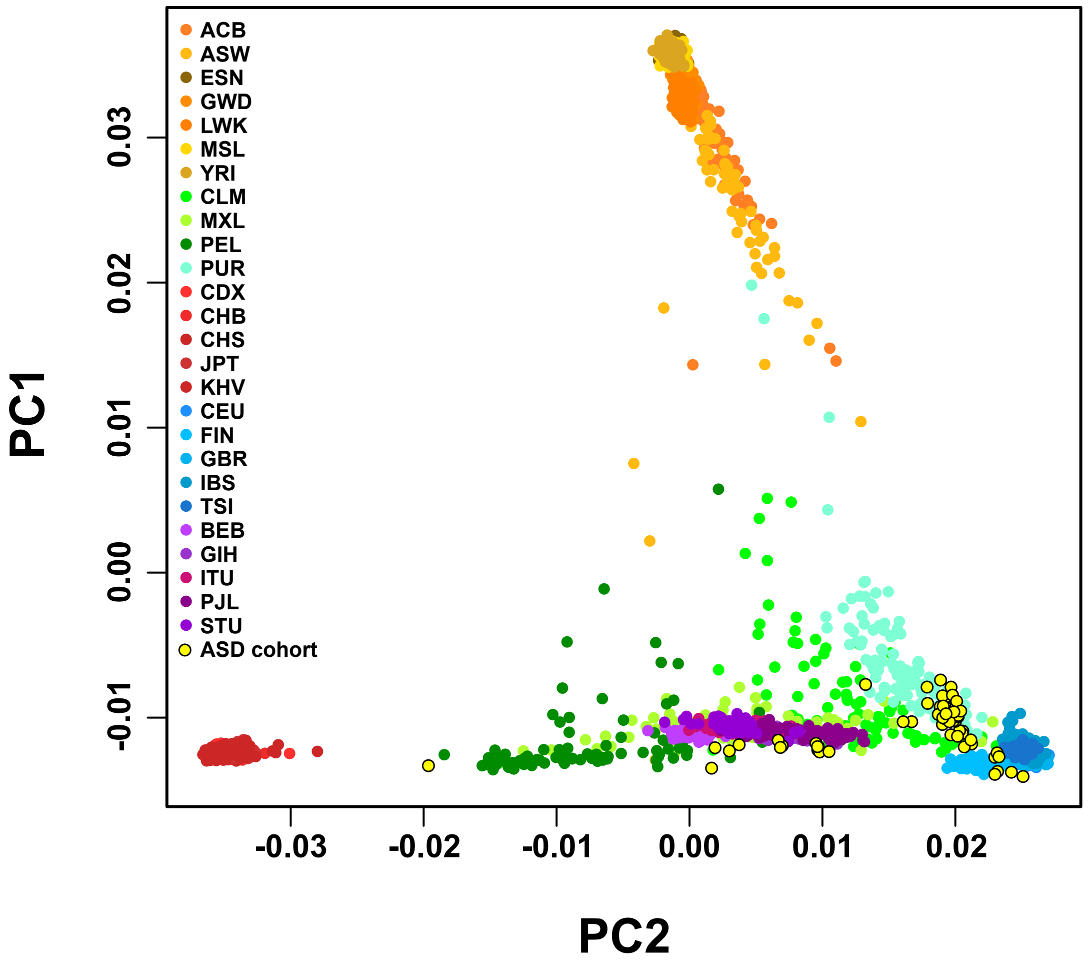

Supplementary Figure 1. Principal component analysis of the consanguineous ASD cohort. Principal component analysis (PCA) of the consanguineous ASD cohort (ASD cohort, bright yellow) combined with 1000G. Population abbreviations are defined in Supplementary Table 14.

Supplementary Figure 2

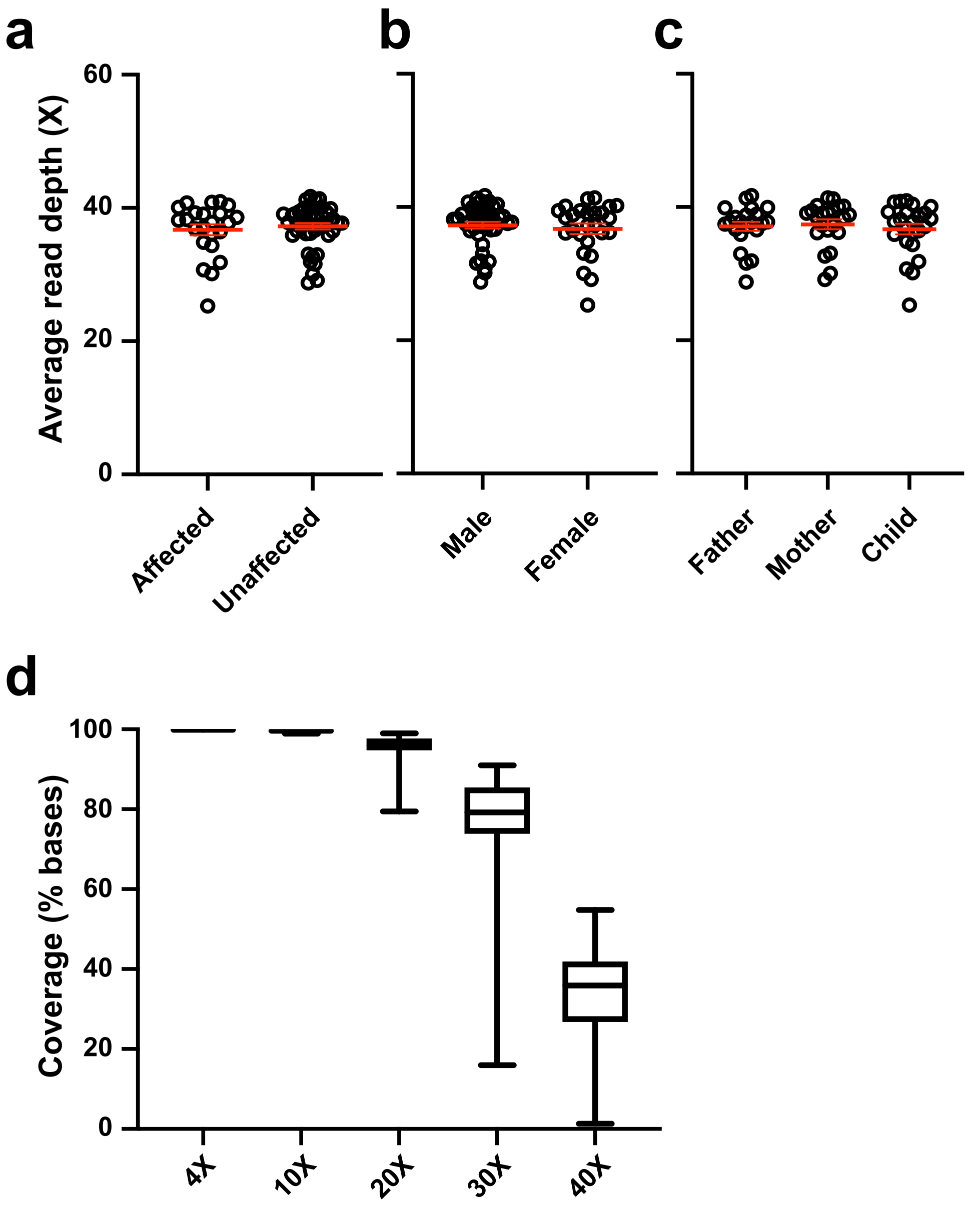

Supplementary Figure 2. Whole genome sequencing coverage statistics. Average read depth per sample was calculated across the cohort (mean ± SEM are shown in red). There were no significant differences in read depth across affection status (N=23 affected, 45 unaffected; *P*=0.5776) (**a**), sex (N=42 male, 26 female; *P*=0.5548) (**b**), or family membership (N=22 father, 22 mother, 24 child; *P*=0.7854) (**c**). The average read depth was 37X across all the plotted categories. Data were analyzed using unpaired t test for (**a**) and (**b**), and ordinary one-way ANOVA followed by Tukey's multiple comparisons test for (**c**). (**d**) The percentage of genomic bases covered at 4X, 10X, 20X, 30X, and 40X read depth was calculated for all samples across the cohort. At each value, the average, minimum, and maximum are plotted. On average, 99.6% and 95.6% of bases were covered at a mean read depth of at least 10X and 20X, respectively.

Supplementary Figure 3

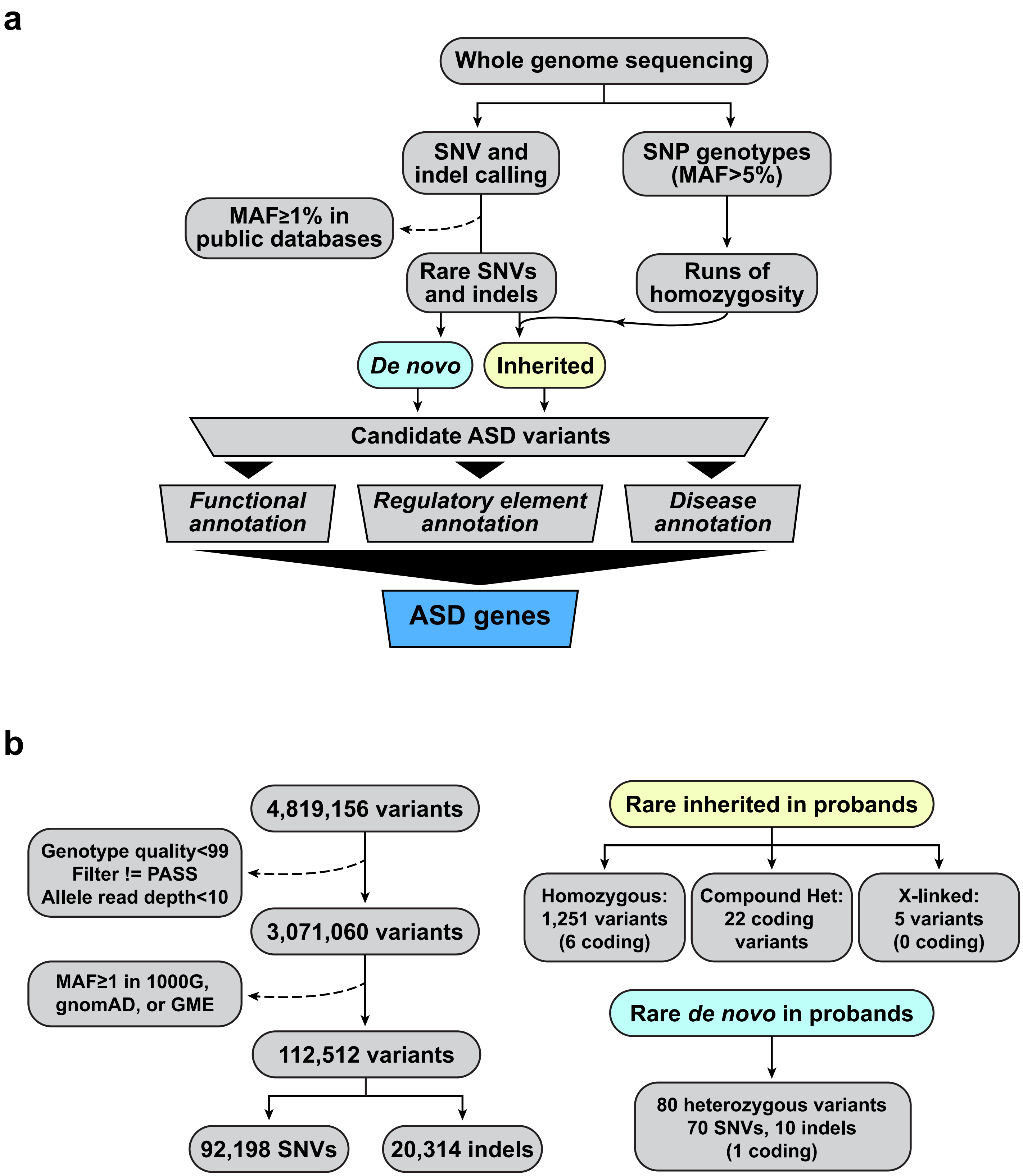

Supplementary Figure 3. Schematic of the data analysis and variant filtration pipelines for the whole genome sequencing data. (**a**) Summary of WGS data analysis for the identification of candidate ASD variants and genes. (**b**) Variant filtration pipeline to identify rare inherited and rare *de novo* variants in affected individuals. At each step, the average number of variants per affected individual is shown.

Supplementary Figure 4

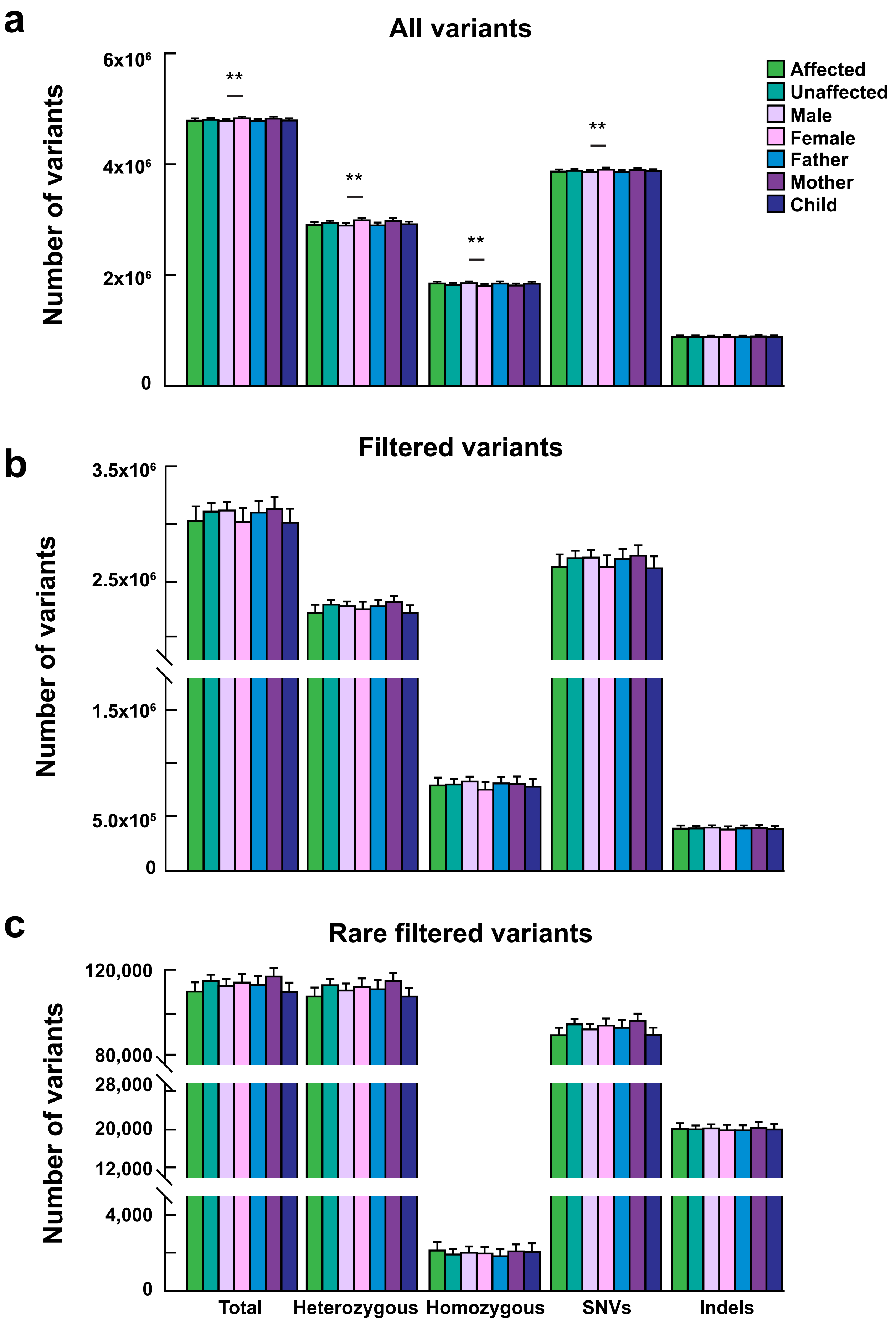

Supplementary Figure 4. Average number of variants identified by whole genome sequencing in the consanguineous ASD cohort. The average number of all variants identified (**a**), all variants that passed quality filters (**b**), and rare (MAF < 1% in all population databases) variants that passed quality filters (**c**). For all variant categories plotted (total, homozygous, heterozygous, SNVs, Indels), there were no significant differences in the number of variants identified across affection status, sex, or family membership in (**b**) or (**c**). In (**a**), for all variant categories plotted, except indels, there was a statistically significant but modest difference (~1-3%) in the number of variants between males and females that can be attributed to the genome size difference (total: ***P*=0.0077; heterozygous: ***P*=0.0035; homozygous: ***P*=0.008; SNVs: ***P*=0.0055). All values are mean ± SEM (N=23 affected, 45 unaffected, 42 male, 26 female, 22 father, 22 mother, 24 child). For affection status and sex, data were analyzed using unpaired t test, and for family membership, data were analyzed using ordinary one-way ANOVA followed by Tukey’s multiple comparisons test.

Supplementary Figure 5

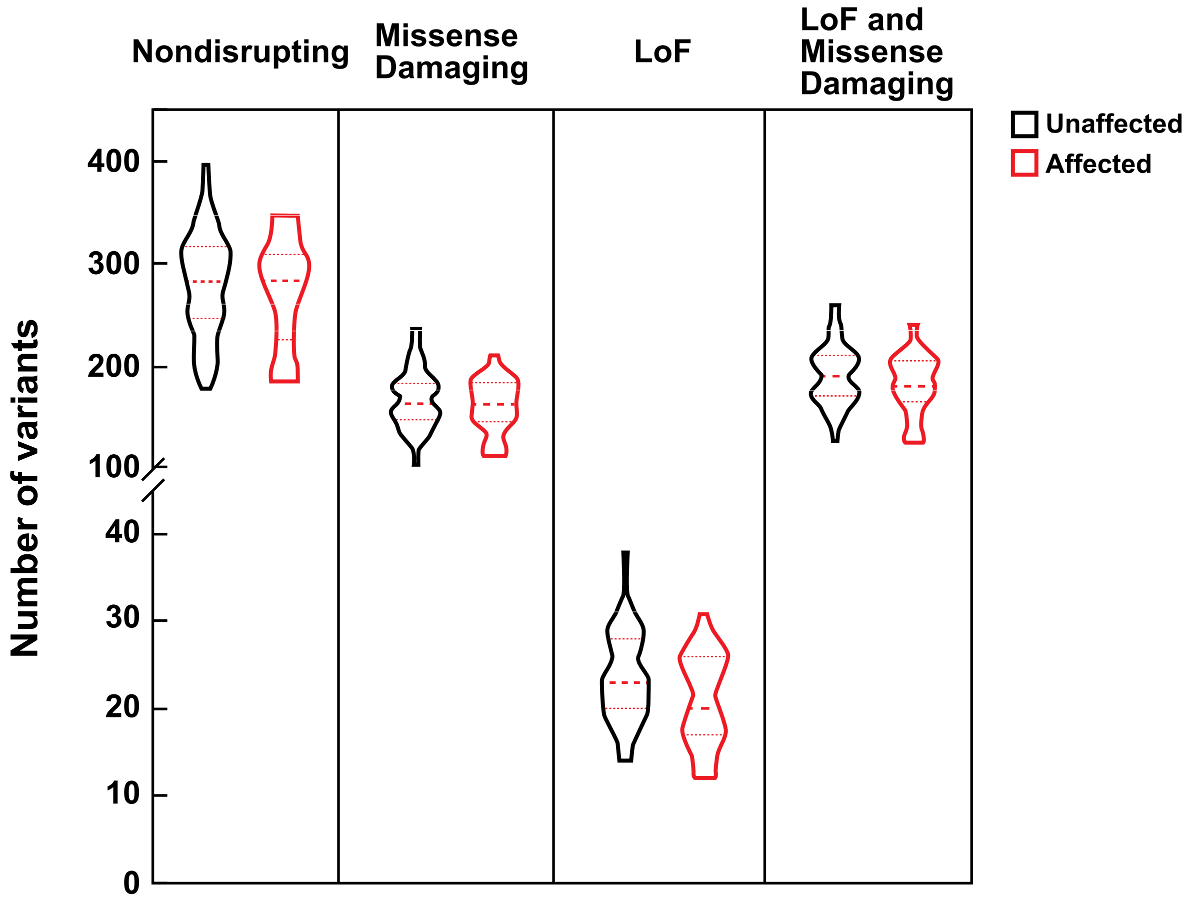

Supplementary Figure 5. Burden analysis of rare variants in the consanguineous ASD cohort. Variant counts in affected (N=23) versus unaffected (N=45) individuals are compared for nondisrupting (P=0.6096), missense damaging (P=0.4114), loss-of-function (LoF) variants (P=0.0601), and for the total missense and LoF variants (P=0.2703). Data were analyzed using unpaired t test. Dotted lines denote 25%, 50%, and 75% marks.

Supplementary Figure 6

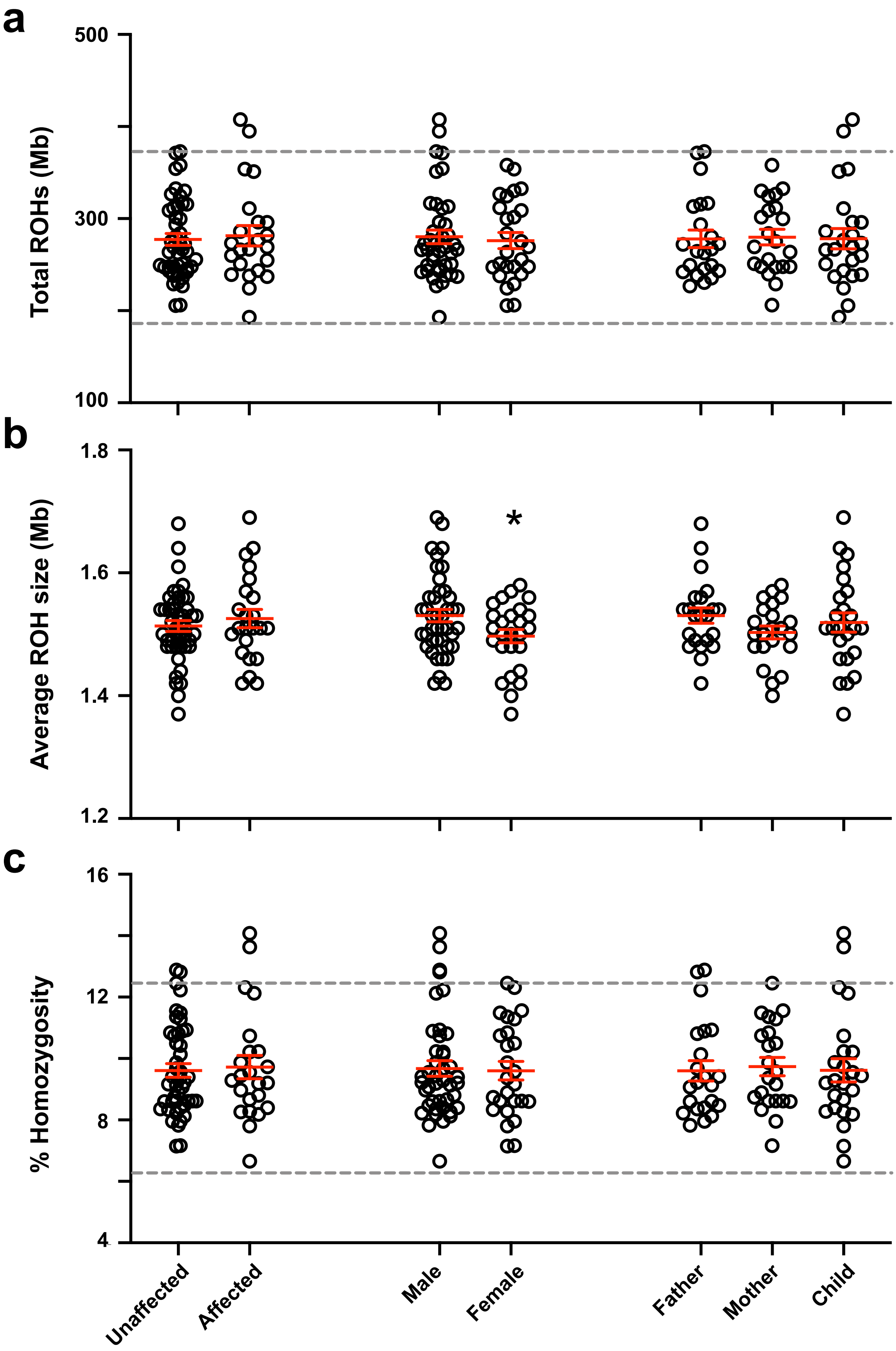

Supplementary Figure 6 continued

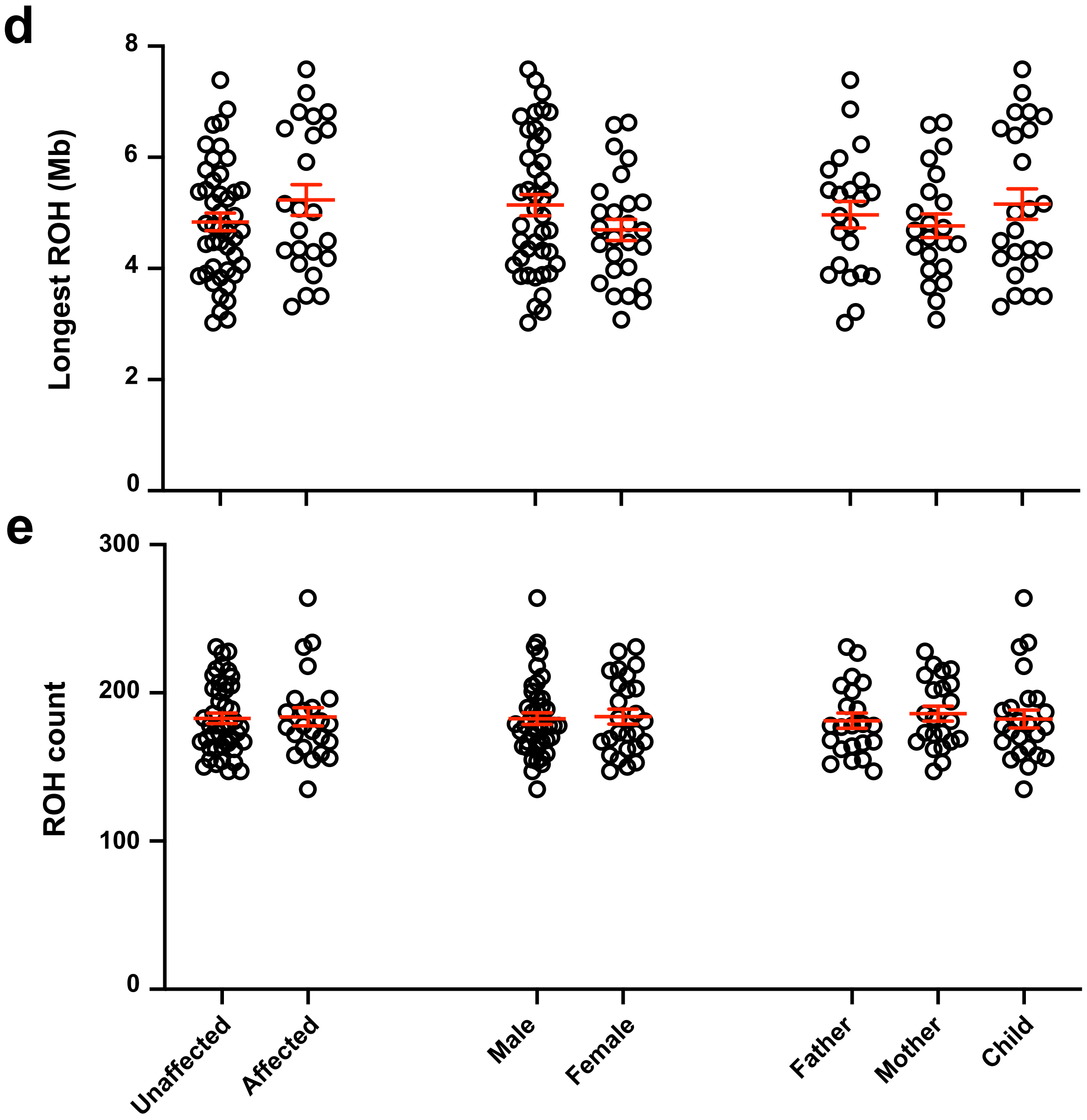

Supplementary Figure 6. Summary statistics for the ROHs identified in the consanguineous ASD cohort. Total length of ROHs (**a**), average ROH size (**b**), percent of homozygosity across the genome (**c**), the size of the longest ROHs identified per individual (**d**), and the number of ROHs identified per individual (**e**), are plotted by affection status, sex, and family membership. The dotted lines in (**a**) and (**c**) denote the expected values for children of second- (top) and third-degree (bottom) relatives. All values are mean ± SEM (N=23 affected, 45 unaffected, 42 male, 26 female, 22 father, 22 mother, 24 child). For affection status and sex, data were analyzed using unpaired t test, and for family membership, data were analyzed using ordinary one-way ANOVA followed by Tukey’s multiple comparisons test. No significant changes were detected across categories, except for a slight increase in average ROH size in males compared to females (N=42 male, 26 female; **P*=0.033).

Supplementary Figure 7

a

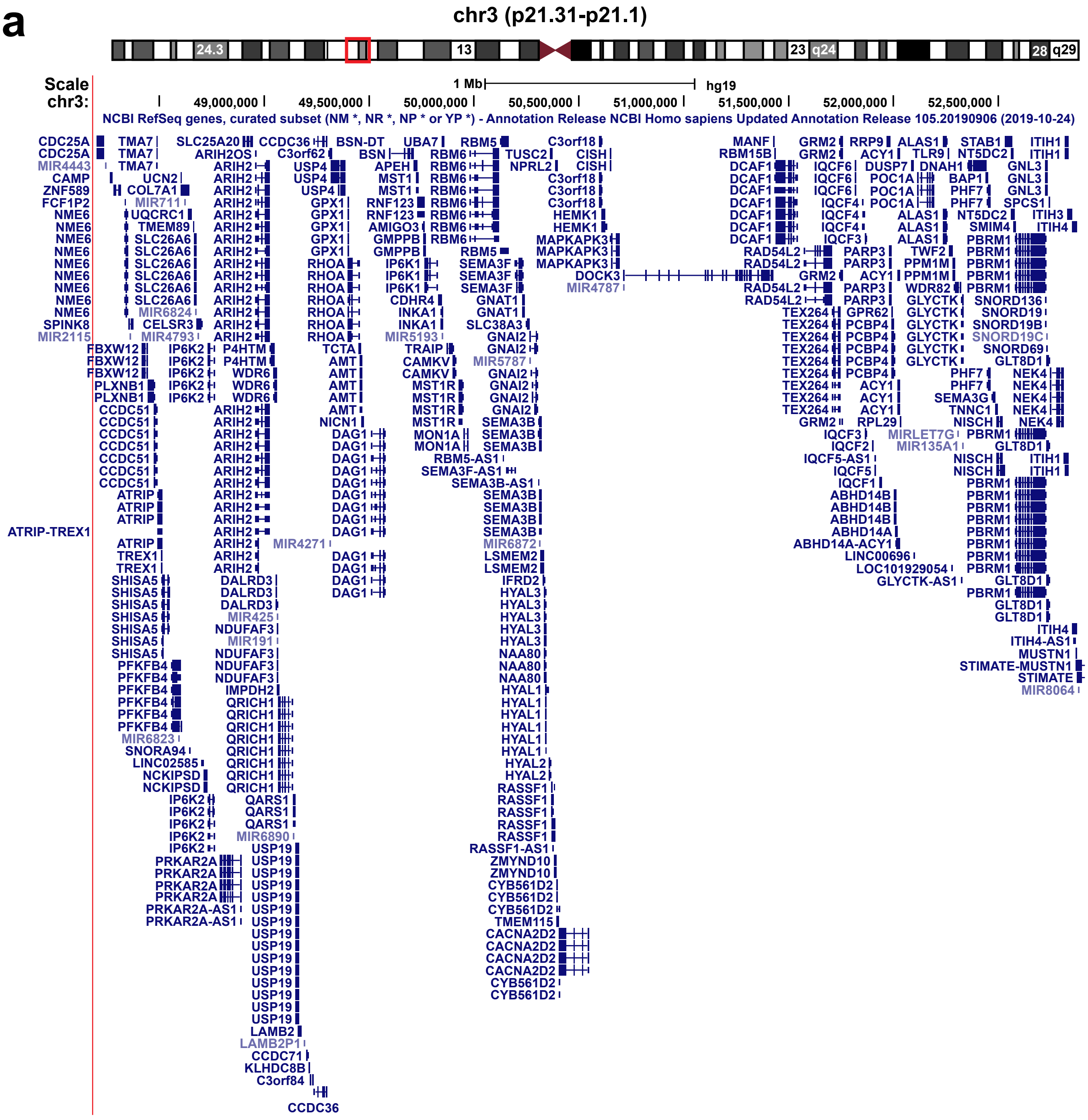

b

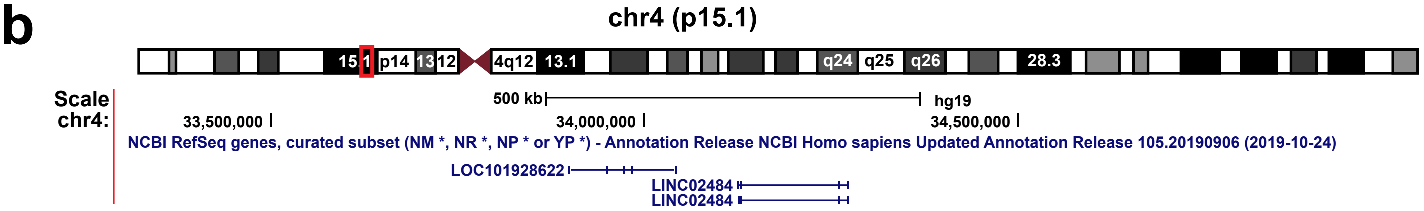

Supplementary Figure 7. The two most common ROHs identified in affected individuals. Genomic maps showing the genes within the two most common ROHs identified in affected individuals (adapted from the UCSC Genome Browser, human reference genome version GRCh37/hg19).

Supplementary Figure 8

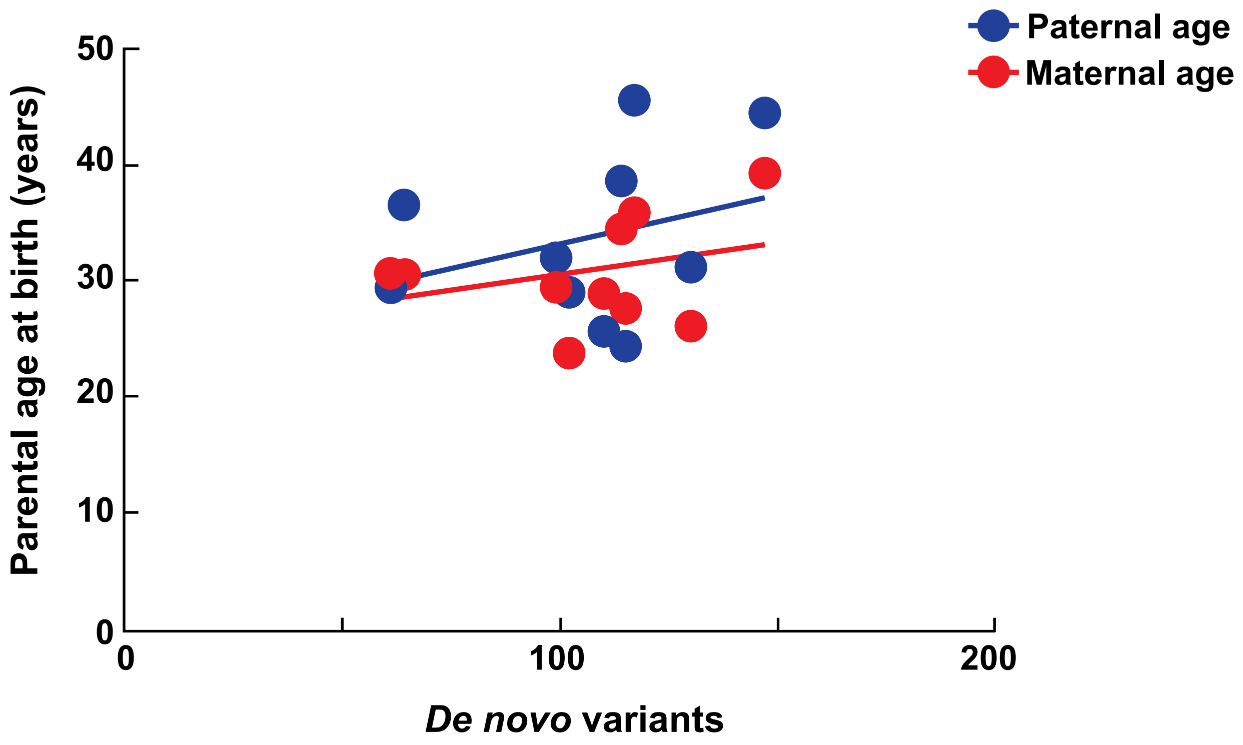

Supplementary Figure 8. The number of rare de novo variants with parental age in the consanguineous ASD cohort. Linear regression analysis showed there were no significant changes in the number of rare *de novo* variants in affected individuals with paternal (N=10; *P*=0.3923, Pearson's r=0.3045) or maternal (N=10; *P*=0.3974, Pearson's r=0.3014) age at birth of the affected offspring.
