# Supplementary material for: Analysis of recent shared ancestry in a familial cohort identifies coding and noncoding autism spectrum disorder variants
Source: NPJ Genom Med. 2022 Feb 21;7:13. doi: 10.1038/s41525-022-00284-2 (PMC8861044; doi:10.1038/s41525-022-00284-2)
Supplement: Supplementary file 3 — Reporting summary [file 41525_2022_284_MOESM3_ESM.pdf]

nature portfolio

Corresponding author(s): Clara Tang
Brian Chung
Kit San Yeung

Last updated by author(s): Oct 28, 2021

# Reporting Summary

Nature Portfolio wishes to improve the reproducibility of the work that we publish. This form provides structure for consistency and transparency in reporting. For further information on Nature Portfolio policies, see our Editorial Policies and the Editorial Policy Checklist.

## Statistics

For all statistical analyses, confirm that the following items are present in the figure legend, table legend, main text, or Methods section.

| n/a | Confirmed | |
|---|---|---|
| ☒ | ☐ | The exact sample size ($n$) for each experimental group/condition, given as a discrete number and unit of measurement |
| ☒ | ☐ | A statement on whether measurements were taken from distinct samples or whether the same sample was measured repeatedly |
| ☒ | ☐ | The statistical test(s) used AND whether they are one- or two-sided<br>*Only common tests should be described solely by name; describe more complex techniques in the Methods section.* |
| ☒ | ☐ | A description of all covariates tested |
| ☒ | ☐ | A description of any assumptions or corrections, such as tests of normality and adjustment for multiple comparisons |
| ☒ | ☐ | A full description of the statistical parameters including central tendency (e.g. means) or other basic estimates (e.g. regression coefficient) AND variation (e.g. standard deviation) or associated estimates of uncertainty (e.g. confidence intervals) |
| ☒ | ☐ | For null hypothesis testing, the test statistic (e.g. $F$, $t$, $r$) with confidence intervals, effect sizes, degrees of freedom and $P$ value noted<br>*Give P values as exact values whenever suitable.* |
| ☒ | ☐ | For Bayesian analysis, information on the choice of priors and Markov chain Monte Carlo settings |
| ☒ | ☐ | For hierarchical and complex designs, identification of the appropriate level for tests and full reporting of outcomes |
| ☒ | ☐ | Estimates of effect sizes (e.g. Cohen's $d$, Pearson's $r$), indicating how they were calculated |

*Our web collection on statistics for biologists contains articles on many of the points above.*

## Software and code

Policy information about availability of computer code

| | |
|---|---|
| Data collection | No software was used in the data collection |
| Data analysis | Reads were aligned to hg19 reference genome using bwa and processed following GATK best practice guidelines including variant calling with HaplotypeCaller. PLINK and peddy was used for sample check and removing duplicate or first-degree related samples. Variants also had to pass Variant Quality Score Recalibration annotated by GATK with a SNP tranche sensitivity threshold of 99.5% and INDEL tranche sensitivity threshold of 99.0%. Gene-specific bioinformatic tools were used for CNV calling for SMN1 and HBA1/HBA2. CNV calling for SMN1 was performed on genome sequencing data by both SMAca and SMNCopyNumberCaller which utilizes differentiating bases between SMN1 and SMN2. CNV calling for HBA1 and HBA2 was performed by NGS4THAL on genome sequencing data. The variants were annotated using ANNOVAR (Build 20200223) with the dbNSFPv4.0a database. |

For manuscripts utilizing custom algorithms or software that are central to the research but not yet described in published literature, software must be made available to editors and reviewers. We strongly encourage code deposition in a community repository (e.g. GitHub). See the Nature Portfolio guidelines for submitting code & software for further information.

## Data

Policy information about availability of data

All manuscripts must include a data availability statement. This statement should provide the following information, where applicable:

- Accession codes, unique identifiers, or web links for publicly available datasets
- A description of any restrictions on data availability
- For clinical datasets or third party data, please ensure that the statement adheres to our policy

The data in this study are available on request from the corresponding authors (K.S.Y, B.H.Y.C., and C.S.M.T). The data are not publicly available as they contain information that could compromise research participant privacy or consent.

# Field-specific reporting

Please select the one below that is the best fit for your research. If you are not sure, read the appropriate sections before making your selection.

☒ Life sciences ☐ Behavioural & social sciences ☐ Ecological, evolutionary & environmental sciences

For a reference copy of the document with all sections, see nature.com/documents/nr-reporting-summary-flat.pdf

# Life sciences study design

All studies must disclose on these points even when the disclosure is negative.

| | |
|---|---|
| Sample size | 1,116 Southern Chinese exome sequencing data samples passed sample QC, whereas for genome sequencing, 427 Southern Chinese and 366 Northern Chinese samples remained for further analysis. The total cohort was recruited into this study for the examination of carrier rate in Southern Chinese population |
| Data exclusions | Duplicate or first-degree relative samples were excluded from this study to prevent double counting carrier rate. |
| Replication | Replication is not relevant to this study. |
| Randomization | Randomization is not relevant to this study. |
| Blinding | Blinding is not relevant to this study. |

# Reporting for specific materials, systems and methods

We require information from authors about some types of materials, experimental systems and methods used in many studies. Here, indicate whether each material, system or method listed is relevant to your study. If you are not sure if a list item applies to your research, read the appropriate section before selecting a response.

## Materials & experimental systems

| n/a | Involved in the study | |
|---|---|---|
| ☒ | ☐ | Antibodies |
| ☒ | ☐ | Eukaryotic cell lines |
| ☒ | ☐ | Palaeontology and archaeology |
| ☒ | ☐ | Animals and other organisms |
| ☐ | ☒ | Human research participants |
| ☒ | ☐ | Clinical data |
| ☒ | ☐ | Dual use research of concern |

## Methods

| n/a | Involved in the study | |
|---|---|---|
| ☒ | ☐ | ChIP-seq |
| ☒ | ☐ | Flow cytometry |
| ☒ | ☐ | MRI-based neuroimaging |

## Human research participants

Policy information about studies involving human research participants

| | |
|---|---|
| Population characteristics | This is a previously reported cohort of 1116 exome sequencing used for secondary finding analysis for pharmacogenetics (PMID: 33600428), and 956 genome sequencing used for studying the genetics of Hirschsprung's disease (PMID: 30217742) |
| Recruitment | Participants were recruited into this study for secondary findings after primary investigations. Duplicate or first-degree relative samples were excluded from this study to prevent double counting carrier rate. |
| Ethics oversight | Ethics approval was granted by the Institutional Review Board, the University of Hong Kong/Hospital Authority Hong Kong |

Ethics oversight | West Cluster (UW12-211, UW12-383, UW 05-282 T/945, UW 12-382, UW 12-469).

Note that full information on the approval of the study protocol must also be provided in the manuscript.
